# Review of Aerosol Transmission of Influenza A Virus

**DOI:** 10.3201/eid1211.060426

**Published:** 2006-11

**Authors:** Raymond Tellier

**Affiliations:** * Hospital for Sick Children, Toronto, Ontario, Canada;; † University of Toronto, Toronto, Ontario, Canada

**Keywords:** Influenza, avian influenza, aerosol transmission, personal protective equipment, pandemic planning, perspective

## Abstract

In theory, influenza viruses can be transmitted through aerosols, large droplets, or direct contact with secretions (or fomites). These 3 modes are not mutually exclusive. Published findings that support the occurrence of aerosol transmission were reviewed to assess the importance of this mode of transmission. Published evidence indicates that aerosol transmission of influenza can be an important mode of transmission, which has obvious implications for pandemic influenza planning and in particular for recommendations about the use of N95 respirators as part of personal protective equipment.

## Abstract 2

Extensive evidence indicates that aerosol transmission of influenza occurs and should be taken into account for pandemic planning.

Concerns about the likely occurrence of an influenza pandemic in the near future are increasing. The highly pathogenic strains of influenza A (H5N1) virus circulating in Asia, Europe, and Africa have become the most feared candidates for giving rise to a pandemic strain.

Several authors have stated that large-droplet transmission is the predominant mode by which influenza virus infection is acquired (*1**–**3*). As a consequence of this opinion, protection against infectious aerosols is often ignored for influenza, including in the context of influenza pandemic preparedness. For example, the Canadian Pandemic Influenza Plan and the US Department of Health and Human Services Pandemic Influenza Plan (*4**,**5*) recommend surgical masks, not N95 respirators, as part of personal protective equipment (PPE) for routine patient care. This position contradicts the knowledge on influenza virus transmission accumulated in the past several decades. Indeed, the relevant chapters of many reference books, written by recognized authorities, refer to aerosols as an important mode of transmission for influenza (*6**–**9*).

In preparation for a possible pandemic caused by a highly lethal virus such as influenza A (H5N1), making the assumption that the role of aerosols in transmission of this virus will be similar to their role in the transmission of known human influenza viruses would seem rational. Because infection with influenza A (H5N1) virus is associated with high death rates and because healthcare workers cannot as yet be protected by vaccination, recommending an enhanced level of protection, including the use of N95 respirators as part of PPE, is important. Following are a brief review of the relevant published findings that support the importance of aerosol transmission of influenza and a brief discussion on the implications of these findings on pandemic preparedness.

## Influenza Virus Aerosols

By definition, aerosols are suspensions in air (or in a gas) of solid or liquid particles, small enough that they remain airborne for prolonged periods because of their low settling velocity. For spherical particles of unit density, settling times (for a 3-m fall) for specific diameters are 10 s for 100 μm, 4 min for 20 μm, 17 min for 10 μm, and 62 min for 5 μm; particles with a diameter <3 μm essentially do not settle. Settling times can be further affected by air turbulence (*10**,**11*).

The median diameters at which particles exhibit aerosol behavior also correspond to the sizes at which they are efficiently deposited in the lower respiratory tract when inhaled. Particles of >6-μm diameter are trapped increasingly in the upper respiratory tract (*12*); no substantial deposition in the lower respiratory tract occurs at >20 μm (*11**,**12*). Many authors adopt a size cutoff of <5 μm for aerosols. This convenient convention is, however, somewhat arbitrary, because the long settling time and the efficient deposition in the lower respiratory tract are properties that do not appear abruptly at a specific diameter value. Certainly, particles in the micron or submicron range will behave as aerosols, and particles >10–20 μm will settle rapidly, will not be deposited in the lower respiratory tract, and are referred to as large droplets (*10**–**12*).

Coughing or sneezing generates a substantial quantity of particles, a large number of which are <5–10 μm in diameter [reviewed in (*10*)]. In addition, particles expelled by coughing or sneezing rapidly shrink in size by evaporation, thereby increasing the number of particles that behave as aerosols. Particles shrunken by evaporation are referred to as droplet nuclei (*10**–**12*). This phenomenon affects particles with a diameter at emission of <20 μm, and complete desiccation would decrease the diameter to a little less than half the initial diameter (*10*). Droplet nuclei are hygroscopic. When exposed to humid air (as in the lungs), they will swell back. One would expect that inhaled hygroscopic particles would be retained in the lower respiratory tract with greater efficiency, and this hypothesis has been confirmed experimentally (*11**,**12*). Because aerosols remain airborne, they can be carried over large distances, which may create a potential for long-range infections. The occurrence of long-range infections is affected by several other factors. These include dilution, the infectious dose, the amount of infectious particles produced, the duration of shedding of the infectious agent, and the persistence of the agent in the environment (*11*). Inferring an absence of aerosols because long-range infections are not frequently observed is incorrect.

Humans acutely infected with influenza A virus have a high virus titer in their respiratory secretions, which will be aerosolized when the patient sneezes or coughs. The viral titer measured in nasopharyngeal washes culminates on approximately day 2 or 3 after infection and can reach up to 10^7^ 50% tissue culture infective dose (TCID_50_)/mL (*13**,**14*). The persistence of the infectivity of influenza virus in aerosols has been studied in the laboratory. In experiments that used homogeneous aerosolized influenza virus suspensions (mean diameter 6 μm), virus infectivity (assessed by in vitro culture) at a fixed relative humidity undergoes an exponential decay; this decay is characterized by very low death rate constants, provided that the relative humidity was in the low range of 15%–40% (*15**,**16*). These results are consistent with those of an older study (admittedly performed in a more rudimentary manner) in which infectious influenza viruses in an aerosol could be demonstrated for up to 24 h by using infection in mice as a detection method, provided that the relative humidity was 17%-24% (*17*). In all these studies, the decay of virus infectivity increased rapidly at relative humidity >40%. The increased survival of influenza virus in aerosols at low relative humidity has been suggested as a factor that accounts for the seasonality of influenza (*15**,**16*). The sharply increased decay of infectivity at high humidity has also been observed for other enveloped viruses (e.g., measles virus); in contrast, exactly the opposite relationship has been shown for some nonenveloped viruses (e.g., poliovirus) (*11**,**15**,**16*).

## Experimental Influenza Infection

Experimental infection studies permit the clear separation of the aerosol route of transmission from transmission by large droplets. Laboratory preparation of homogeneous small particle aerosols free of large droplets is readily achieved (*13**,**18*). Conversely, transmission by large droplets without accompanying aerosols can be achieved by intranasal drop inoculation (*13*).

Influenza infection has been documented by aerosol exposure in the mouse model, the squirrel monkey model, and human volunteers (*12**,**13**,**17**–**19*). Observations made during experimental infections with human volunteers are particularly interesting and relevant. In studies conducted by Alford and colleagues (*18*), volunteers were exposed to carefully titrated aerosolized influenza virus suspensions by inhaling 10 L of aerosol through a face mask. The diameter of the aerosol particles was 1 μm–3 μm. Demonstration of infection in participants in the study was achieved by recovery of infectious viruses from throat swabs, taken daily, or by seroconversion, i.e., development of neutralizing antibodies. The use of carefully titrated viral stocks enabled the determination of the minimal infectious dose by aerosol inoculation. For volunteers who lacked detectable neutralizing antibodies at the onset, the 50% human infectious dose (HID_50_) was 0.6–3.0 TCID_50_, if one assumes a retention of 60% of the inhaled particles (18). In contrast, the HID_50_ measured when inoculation was performed by intranasal drops was 127–320 TCID_50_ (*13*). Additional data from experiments conducted with aerosolized influenza virus (average diameter 1.5 μm) showed that when a dose of 3 TCID_50_ was inhaled, ≈1 TCID_50_ only was deposited in the nose (*12*). Since the dose deposited in the nose is largely below the minimal dose required by intranasal inoculation, this would indicate that the preferred site of infection initiation during aerosol inoculation is the lower respiratory tract. Another relevant observation is that whereas the clinical symptoms initiated by aerosol inoculation covered the spectrum of symptoms seen in natural infections, the disease observed in study participants infected experimentally by intranasal drops was milder, with a longer incubation time and usually no involvement of the lower respiratory tract (*13**,**20*). For safety reasons, this finding led to the adoption of intranasal drop inoculation as the standard procedure in human experimental infections with influenza virus (*13*).

Additional support for the view that the lower respiratory tract (which is most efficiently reached by the aerosol route) is the preferred site of infection is provided by studies on the use of zanamivir for prophylaxis. In experimental settings, intranasal zanamivir was protective against experimental inoculation with influenza virus in intranasal drops (*21*). However, in studies on prophylaxis of natural infection, intranasally applied zanamivir was not protective (*22*), whereas inhaled zanamivir was protective in one study (*23*) and a protective effect approached statistical significance in another study (*22*). These experiments and observations strongly support the view that many, possibly most, natural influenza infections occur by the aerosol route and that the lower respiratory tract may be the preferred site of initiation of the infection.

## Epidemiologic Observations

In natural infections, the postulated modes of transmission have included aerosols, large droplets, and direct contact with secretions or fomites because the virus can remain infectious on nonporous dry surfaces for <48 hours (*24*). Because in practice completely ruling out contributions of a given mode of transmission is often difficult, the relative contribution of each mode is usually difficult to establish by epidemiologic studies alone. However, a certain number of observations are consistent with and strongly suggestive of an important role for aerosol transmission in natural infections, for example the "explosive nature and simultaneous onset [of disease] in many persons" (*9*), including in nosocomial outbreaks (*25*). The often-cited outbreak described by Moser et al. on an airplane with a defective ventilation system is best accounted for by aerosol transmission (*26*). Even more compelling were the observations made at the Livermore Veterans Administration Hospital during the 1957–58 pandemic. The study group consisted of 209 tuberculous patients confined during their hospitalization to a building with ceiling-mounted UV lights; 396 tuberculous patients hospitalized in other buildings that lacked these lights constituted the control group. Although the study group participants remained confined to the building, they were attended to by the same personnel as the control group, and there were no restrictions on visits from the community. Thus, it was unavoidable at some point that attending personnel and visitors would introduce influenza virus in both groups. During the second wave of the pandemic, the control group and the personnel sustained a robust outbreak of respiratory illness, shown retrospectively by serology to be due to the pandemic strain influenza A (H2N2), whereas the group in the irradiated building remained symptom free. The seroconversion rate to influenza A (H2N2) was 19% in the control group, 18% in personnel, but only 2% in the study group (*27**,**28*).

Whereas UV irradiation is highly effective in inactivating viruses in small-particle aerosols, it is ineffective for surface decontamination because of poor surface penetrations. It is also ineffective for large droplets because the germicidal activity sharply decreases as the relative humidity increases (*28*). Furthermore, because the installation of UV lights was set up in such a way as to decontaminate the upper air of rooms only, large droplets would not have been exposed to UV, whereas aerosols, carried by thermal air mixing, would have been exposed (*27**,**28*). So in effect in this study only the aerosol route of infection was blocked, and this step alone achieved near complete protection.

The converse occurrence, blocking only the large droplet and fomites routes in natural infections, can be inferred from the studies on the use of zanamivir for prophylaxis described previously. In experimental settings, intranasally applied zanamivir was protective against an experimental challenge with influenza by intranasal drops (*21*). However, in studies on prophylaxis of natural disease, intranasal zanamivir was not protective (*22*), which leads to the conclusion that natural infection can occur efficiently by a route other than large droplets or fomites. As noted above, inhaled zanamivir was significantly protective (*22**,**23*).

## Discussion and Implications for Infection Control during Influenza A (H5) Pandemic

In principle, influenza viruses can be transmitted by 3 routes: aerosols, large droplets, and direct contact with secretions (or with fomites). These 3 routes are not mutually exclusive and, as noted above, may be difficult to disentangle in natural infections.

For the purpose of deciding on the use of N95 respirators in a pandemic, showing that aerosol transmission occurs at appreciable rates is sufficient. Evidence supporting aerosol transmission, reviewed above, appears compelling. Despite the evidence cited in support of aerosol transmission, many guidelines or review articles nevertheless routinely state that "large droplets transmission is thought to be the main mode of influenza transmission" (or similar statements) without providing supporting evidence from either previously published studies or empirical findings. Despite extensive searches, I have not found a study that proves the notion that large-droplets transmission is predominant and that aerosol transmission is negligible (or nonexistent). Reports on many outbreaks suggest that influenza aerosols are rapidly diluted because long-range infections occur most spectacularly in situations of crowding and poor ventilation (*25**,**26*). However, even if long-range infections do not readily occur when sufficient ventilation exists, this does not rule out the presence at closer range of infectious particles in the micron or submicron range, against which surgical masks would offer little protection (*29**,**30*). Many infection control practitioners have argued that the introduction of large-droplets precautions in institutions has proven sufficient to interrupt influenza outbreaks and therefore that aerosol transmission appears negligible. This evidence is, unfortunately, inconclusive because of several confounding or mitigating factors. First, unless precise laboratory diagnosis is obtained, respiratory syncytial virus outbreaks can be mistaken for influenza outbreaks (*9*), which would artificially increase the perceived "effectiveness" of large-droplets precautions against influenza. Second, serologic studies are often not conducted, and therefore asymptomatic infections are not documented (among healthcare workers a large fraction of influenza infections are asymptomatic or mistaken for another disease [31]). Third, since we are in an interpandemic period and the viruses currently circulating have been drifting from related strains for decades, we all have partial immunity against these viruses, immunity that is further boosted in vaccinated healthcare workers. It has even been argued that after several decades of circulation the current human influenza viruses are undergoing gradual attenuation (*32*). Finally, surgical masks (used in large-droplets precautions) do not offer reliable protection against aerosols, but they nevertheless have a partially protective effect, which further confuses the issue (*29**,**30*).

In contrast, the situation with a pandemic strain of influenza A (H5) would become only too clear because no one would have any degree of immunity against such a virus, vaccines would not be available for months, and these viruses would likely be highly virulent. Even though efficient human-to-human transmission of the A (H5N1) virus has not yet been observed (by any mode), transmission of influenza A (H5N1) by aerosols from geese to quails has been demonstrated in the laboratory (*33*). Thus, even in the current incarnation of A (H5N1), infection by the virus can generate aerosols that are infectious for highly susceptible hosts. As far as we know, 1 of the main blocks to efficient human-to-human transmission of influenza A (H5N1) is the virus's current preference for specific sialic acid receptors. The current strains still prefer α-2,3–linked sialic acids, which is typical of avian influenza viruses, whereas human influenza viruses bind preferentially to α-2,6–linked sialic acids (*34**–**36*). In all likelihood, 1 of the mutations required for influenza A (H5N1) to give rise to a pandemic strain would be to change its receptor affinity to favor the α-2,6–linked sialic acids. For the influenza A (H1N1) pandemic strain of 1918, this change required only 1 or 2 amino acid substitutions (*36*). Once a highly transmissible strain of influenza A (H5) has arisen, it will likely spread in part by aerosols, like other human influenza viruses.

Recent studies have shown that whereas epithelial cells in the human respiratory tract express predominantly the α-2,6 sialic acid receptor, cells expressing the α-2,3 receptor were detected only occasionally in the upper respiratory tract; however, measurable expression of α-2,3–linked sialic acid receptors was found in some cells in the alveolar epithelium and at the junction of alveolus and terminal bronchiole (*35*). Binding of influenza A (H5N1) virus can be demonstrated in human tissue sections from the respiratory tract in a distribution corresponding to that of the α-2,3 receptors in the respiratory tract (*34**,**35*). This pattern of virus binding correlates well with autopsy findings, which show extensive alveolar damage (*34**,**37*), and also correlates well with the observation that recovery of the A (H5N1) virus is much more difficult from nasal swabs than from throat swabs (*37*). Thus, in the respiratory system the current strains of A (H5N1) appear to infect mostly (perhaps exclusively) the lower respiratory tract. If that is indeed the case, it in turn suggests that human cases of avian influenza were acquired by exposure to an aerosol, since large droplets would not have delivered the virus to the lower respiratory tract. (Another hypothesis might be gastrointestinal infection, followed by viremia and dissemination, but not all patients have gastrointestinal symptoms [37]). Given the strong evidence for aerosol transmission of influenza viruses in general, and the high lethality of the current strains of avian influenza A (H5N1) (*37*), recommending the use of N95 respirators, not surgical masks, as part of the protective equipment seems rational.

Several infection control guidelines for influenza have recently been published, some specifically aimed at the current strains of A (H5N1), others as part of more comprehensive pandemic plans that address the emergence not only of a pandemic form of A (H5) but also of other types of pandemic influenza viruses. Even though to date human-to-human transmission of A (H5N1) remains very inefficient, the high lethality of the infection and potential for mutations call for prudence. The use of N95 respirators is included in the 2004 recommendations of the Centers for Disease Control and Prevention for healthcare workers who treat patients with known or suspected avian influenza (*38*). The World Health Organization's current (April 2006) guidelines for avian influenza recommend the use of airborne precautions when possible, including the use of N95 respirators when entering patients' rooms (*39*).

Currently, several pandemic plans differ considerably in their recommendations for infection control precautions and PPE. The current version of the Canadian pandemic plan recommends surgical masks only, disregarding data that support the aerosol transmission of influenza (*4*). The US pandemic plans (*5*) and the British plans, from both the National Health Service (available from http://www.dh.gov.uk/PublicationsAndStatistics/Publications/PublicationsPolicyAndGuidance/

PublicationsPolicyAndGuidanceArticle/fs/en?CONTENT_ID=4121735&chk=Z6kjQY) and the Health Protection Agency (http://www.hpa.org.uk/infections/topics_az/influenza/pandemic/pdfs/HPAPandemicplan.pdf), acknowledge the contribution of aerosols in influenza but curiously recommend surgical masks for routine care; the use of N95 respirators is reserved for protection during "aerosolizing procedures" (*5**,**40*). These recommendations fail to recognize that infectious aerosols will also be generated by coughing and sneezing. The Australian Management Plan for Pandemic Influenza (June 2005) recommends N95 respirators for healthcare workers (http://www.health.gov.au/internet/wcms/Publishing.nsf/Content/phd-pandemic-plan.htm), and in France, the Plan gouvernemental de prévention et de lutte <<Pandémie grippale>>(January 2006) recommends FFP2 respirators (equivalent to N95 respirators) (http://www.splf.org/s/IMG/pdf/plan-grip-janvier06.pdf). Given the scientific evidence that supports the occurrence of aerosol transmission of influenza, carefully reexamining current recommendations for PPE equipment would appear necessary.
